# Policy to implementation: evidence-based practice in community mental health – study protocol

**DOI:** 10.1186/1748-5908-8-38

**Published:** 2013-03-24

**Authors:** Rinad S Beidas, Gregory Aarons, Frances Barg, Arthur Evans, Trevor Hadley, Kimberly Hoagwood, Steven Marcus, Sonja Schoenwald, Lucia Walsh, David S Mandell

**Affiliations:** 1 Department of Psychiatry, University of Pennsylvania Perelman School of Medicine, 3535 Market Street, 3rd floor, Philadelphia, PA, 19104, USA; 2 Department of Psychiatry, University of California San Diego, San Diego, CA, USA; 3 Department of Family Medicine and Community Health, University of Pennsylvania Perelman School of Medicine, Philadelphia, PA, USA; 4 Department of Behavioral Health and Intellectual DisAbility Services, Philadelphia, PA, USA; 5 Department of Psychiatry, New York University, New York, NY, USA; 6 Center for Health Equity Research and Promotion, Philadelphia Veterans Affairs Medical Center; School of Social Policy and Practice, University of Pennsylvania, Philadelphia, PA, USA; 7 Department of Psychiatry and Behavioral Sciences, Medical University of South Carolina, Charleston, SC, USA

**Keywords:** Evidence-based practice, Community mental health, Policy, Implementation, Fidelity, Organizational variables

## Abstract

**Background:**

Evidence-based treatments (EBTs) are not widely available in community mental health settings. In response to the call for implementation of evidence-based treatments in the United States, states and counties have mandated behavioral health reform through policies and other initiatives. Evaluations of the impact of these policies on implementation are rare. A systems transformation about to occur in Philadelphia, Pennsylvania, offers an important opportunity to prospectively study implementation in response to a policy mandate.

**Methods/design:**

Using a prospective sequential mixed-methods design, with observations at multiple points in time, we will investigate the responses of staff from 30 community mental health clinics to a policy from the Department of Behavioral Health encouraging and incentivizing providers to implement evidence-based treatments to treat youth with mental health problems. Study participants will be 30 executive directors, 30 clinical directors, and 240 therapists. Data will be collected prior to the policy implementation, and then at two and four years following policy implementation. Quantitative data will include measures of intervention implementation and potential moderators of implementation (*i.e.*, organizational- and leader-level variables) and will be collected from executive directors, clinical directors, and therapists. Measures include self-reported therapist fidelity to evidence-based treatment techniques as measured by the Therapist Procedures Checklist-Revised, organizational variables as measured by the Organizational Social Context Measurement System and the Implementation Climate Assessment, leader variables as measured by the Multifactor Leadership Questionnaire, attitudes towards EBTs as measured by the Evidence-Based Practice Attitude Scale, and knowledge of EBTs as measured by the Knowledge of Evidence- Based Services Questionnaire. Qualitative data will include semi-structured interviews with a subset of the sample to assess the implementation experience of high-, average-, and low-performing agencies. Mixed methods will be integrated through comparing and contrasting results from the two methods for each of the primary hypotheses in this study.

**Discussion:**

Findings from the proposed research will inform both future policy mandates around implementation and the support required for the success of these policies, with the ultimate goal of improving the quality of treatment provided to youth in the public sector.

## Background

Evidence-based treatments (EBTs) are treatments that have been evaluated scientifically and show evidence of efficacy [1]. Despite well-established evidence of EBTs for youth with psychosocial difficulties [1], it takes up to 17 years for these treatments to make their way into community settings [2]. In response to the call for implementation of EBTs [3], systems have mandated behavioral health reform [4] through policies and other initiatives. Evaluations of the impact of these policies on implementation are rare [5]. While policies may be important drivers of implementation, they are likely necessary but not sufficient. In particular, organization- and leader-level variables may moderate the relationship between policy and implementation.

A burgeoning literature has applied evidence from organizational theory to mental health service organizations [6] and found that specific organizational level constructs influence adoption and sustainability of new practices. Constructs of particular interest include organizational culture, organizational climate, and implementation climate. Organizational culture is defined as shared beliefs and expectations of a work environment, whereas organizational climate is defined as shared perceptions about the work environment’s impact on worker well-being [7]. Organizational climate has been associated with both implementation and youth outcomes [8]. Even more compelling, interventions that improve organizational climate can improve implementation of EBTs in the community [9].

Distinct from organizational climate, implementation climate is defined as staff beliefs regarding the degree to which an innovation is expected, rewarded and supported by their organization [10]. Little empirical measurement of implementation climate has been conducted in mental health services research [11], but research from other disciplines suggests that it is highly predictive of implementation [12].

Leadership may also drive implementation of EBTs, although few studies have examined its effects. One model of effective leadership [13] comprises five factors: individual consideration (consideration for each employee’s contributions and needs), intellectual stimulation (potential to stimulate employee thinking), inspirational motivation (potential to inspire and motivate employees), idealized influence attributed (ability to instill pride in employees), and idealized influence behavior (ability to instill values, beliefs, purpose, and mission in employees) [13]. Preliminary research on the associations among leadership and organizational variables has found that high-quality leadership is important in times of system change and may reduce poor organizational climate and subsequent staff turnover [4]. High-quality leadership is also associated with better staff attitudes towards adopting EBTs [14]. It is therefore critical to investigate if high-quality leadership and characteristics of leaders (*e.g*., attitudes) predict more successful implementation of child EBTs in the face of a policy mandate.

### Systems transformation in Philadelphia

The City of Philadelphia’s Department of Behavioral Health and Intellectual DisAbility Services (DBHIDS) is committed to transforming their public system into one that is evidence-based for both adults and children. The behavioral health care of Medicaid-enrolled individuals with Philadelphia is managed through Community Behavioral Health (CBH), a quasi-governmental administrative service organization. Since 2007, DBHIDS has engaged in pilot EBT implementation projects in the public mental health system. In 2012, the Commissioner of DBHIDS (AE), assembled the Evidence-Based Practice & Innovation Center (EPIC), a task force of expert academics and leaders at DBHIDS, to develop a coordinated approach and centralized infrastructure that supports providers in implementing, utilizing, and sustaining EBTs. The contributions of EPIC will be phased. The first phase entails compiling lessons learned from pilot EBT implementation projects, engaging community stakeholders, and selection of an implementation framework to guide the building of the infrastructure. Once established, EPIC will provide support in a number of areas, including: system-wide promotion of evidence-based principles, building of provider capacity for EBTs, operational support, developing an infrastructure for training and ongoing support, and potentially implementation of financing models to promote sustainability (*e.g*., enhanced rates for implementation of EBTs). Currently, EPIC is in the first phase; the completion of the process and infrastructure are anticipated in the next fiscal year. Based on the activities of EPIC, a recommendation will be made by the regulating body, DBHIDS, on implementation of EBTs; we operationally define this recommendation as a policy mandate.

The systems transformation about to occur in Philadelphia offers a rare and important opportunity to prospectively study implementation in response to a policy mandate from inception to implementation. The objective of the proposed research is to observe how community mental health providers (CMHPs) respond to a system-level policy designed to increase implementation of EBTs for youth and adults with mental health difficulties, and to investigate if organizational and leadership characteristics moderate the association between policy and implementation. Specifically, the overall objectives of the study are to answer the following questions within CMHPs: Does a policy mandate impact implementation of EBTs in community mental health?; Do organizational- and leader-level variables moderate the relationship between policy and implementation of EBTs?; What factors characterize the differences among providers with low, average, and high implementation?

### Conceptual framework and causal model

**Figure 1:**
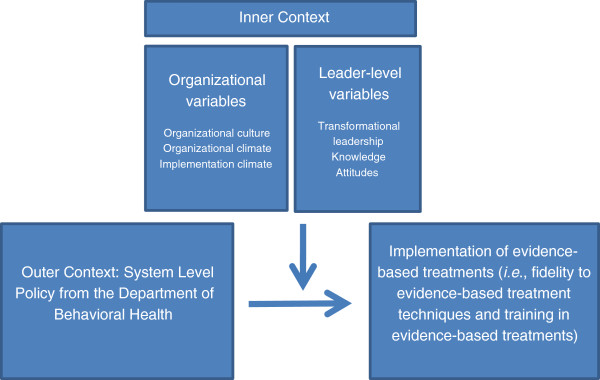
Causal model.

The proposed research activities are based on the conceptual model of EBT implementation in public service sectors proposed by Aarons and colleagues [15]. This four-phase multi-level ecological model of the implementation process for EBTs in public sector settings is both a process and explanatory framework. The process steps include exploration, preparation, implementation, and sustainment (the EPIS model). Within each phase, particular contextual variables are relevant to the outer (external to the provider at the service system level) or inner (internal to the provider implementing an EBT) context. We will prospectively measure a subset of variables from the EPIS model to examine their association with implementation effectiveness in CMHPs serving youth (Figure 1). The current study will assess: the impact of an outer context change (*i.e*., policy) on implementation; and how inner context variables, organizational and leader characteristics, moderate the relationship between policy and implementation.

All three aims draw on the following causal model. Policy, an outer context variable, is defined as a recommendation and support made by a regulating body to promote implementation of EBTs. In this causal model, policy is directly related to the dependent variable, implementation of EBTs [16]. Inner context variables, specifically organizational- and leader-level variables, are hypothesized to moderate this association. In public health, the impact of policy is well documented, as many studies have shown that seatbelt usage can prevent injury and death. Policies have been enacted to require seatbelt usage and have resulted in reduced mortality and injury [17]. However, little is known about how policy impacts and interacts with organizational characteristics to affect provider behavior change [16]. We hypothesize that a policy mandate that is made by a city regulating agency (DBHIDS) will potentially have a powerful impact on provider behavior change.

## Methods/design

### Aim 1: to evaluate child-serving CMHPs response to a system-wide policy mandating implementation of EBTs

Aim 1 tests the causal relationship: Does a system-level policy impact implementation of EBTs in child-serving CMHPs?

### Participants

There are over 100 CMHPs in Philadelphia that provide outpatient services to youth (information provided through personal communication, Community Behavioral Health, 2012). We will enroll at least 30 CMHPs; enrolled CMHPs will serve a combined total of at least 80% of youth receiving publicly-funded mental health services in Philadelphia. In each agency, we will recruit the executive director, clinical director, and 80% of the clinicians (estimated 8 to 10) per agency. This will produce a total sample of 30 provider organizations, 30 executive directors, 30 clinical directors, and 240 to 300 clinicians.

### Procedure

We will prospectively measure CMHP response to the policy generated from the DBHIDS task force. Response is operationally defined as implementation of child EBTs. In order to have multiple indicators of implementation [18], we have defined implementation in two ways. The primary outcome is clinician fidelity to techniques used in child EBTs. The secondary outcome is more proximal to the policy change and the reach of EBTs at the clinician level. Thus, this includes number of clinicians trained in a specific EBT at any given data collection point. We will measure these outcome variables three times over five years in enrolled CMHPs.

### Measures

#### Dependent variable: implementation

In Aim 1, the implementation outcomes include fidelity to EBT techniques and number of clinicians trained in EBTs.

#### Fidelity

We selected fidelity, ‘the degree to which an intervention was implemented as it was prescribed’ [18], as the primary implementation outcome given its documented association with youth outcomes [19]. A number of other implementation outcomes could have been selected, such as acceptability, feasibility and adoption [18]. Ultimately, we decided to focus on fidelity because it is most proximal to youth outcomes, the desired end goal of implementing EBTs. Fidelity will be measured three times using self-reported clinician fidelity to EBTs and brief observation.

#### Therapist Procedures Checklist-Revised (TPC-R)

The TPC-R [20] is a 62-item psychometrically validated self-report clinician technique checklist that assesses components of EBTs used in session that cut across the most widely used modalities (cognitive, behavioral, family and psychodynamic). Factor analysis has confirmed the four-factor structure, test-retest reliability is strong, and the instrument is sensitive to within-therapist changes in technique use across child clients [20].

#### Therapy Procedures Observational Coding System – Strategies (TPOCS-S) [21]

Because self-reported fidelity often does not match actual behavior [22], and to avoid demand characteristics on reporting the use of EBTs, we will use brief observation to corroborate clinician self-report. We will randomly select 10% of therapy sessions in one week of a subset of the clinicians enrolled (n = 120) for observation. We will use the TPOCS-S to code for presence or absence of EBT techniques and intensity to which therapists use these strategies in session. The TPOCS–S is an observational measure of youth psychotherapy procedures. The TPOCS–S shows good inter-rater reliability and its five subscales (*e.g*., Behavioral, Cognitive, Psychodynamic, Client-Centered, Family) show good internal consistency and validity [21].

One of the challenges to measuring fidelity in multiple agencies is that agencies may select to receive training and implement different EBTs based on the populations that they serve. Therefore, it is necessary either to use different fidelity measures, many of which are not validated, across agencies based on which EBT they implement, or to use a general validated measure that allows for identification of common elements across EBTs. We selected the TPC-R because it is a psychometrically validated general fidelity measure that identifies fidelity to techniques (*e.g*., cognitive restructuring) used by the clinician that are non-specific to a particular treatment. We also elected to include an observational measure of practice to ensure that self-report is accurate.

#### Training

Our secondary implementation outcome comprises a numerical count of clinicians trained in EBTs. We will gather this information by asking clinicians to complete a brief survey regarding their training in EBTs selected by the task force to be implemented. We will also provide a list of EBTs and ask if they have been trained in any of the modalities, or used them with one or more clients in the past year.

#### Aim 2: to examine organization- and leader-level variables as moderators of implementation of EBTs

Recent research suggests that organizational- [8] and leader-level variables may be important proximal predictors of implementation of EBTs. Because an outer context policy is a distal predictor of implementation, inner context variables likely play an important role in implementation success. We will examine organizational- and leadership-level variables as moderators of the association between policy and implementation.

#### Participants

See Aim 1. To measure organizational level constructs such as climate and culture, 80% of clinicians from each CMHP will complete the measures described below. We will also collect relevant information from executive and clinical directors.

#### Procedure

In addition to the information gathered in Aim 1, we will prospectively measure organizational- and leader-level variables in CMHPs. Organizational variables include organizational culture, organizational climate, and implementation climate. Leader-level variables include transformational leadership, leader knowledge of EBTs, and attitudes toward EBTs. We will collect leadership data on both the executive director and the clinical director.

### Measures

#### Organizational climate and culture

##### Organizational Social Context Measurement System (OSC)

The OSC [6] is a 105-item measure of the social context of mental health and social services organizations. The OSC measures organizational culture, organizational climate, and work attitudes. We considered a number of measures that assess organizational variables (*e.g*., Organizational Readiness for Change [23], Organizational Readiness for Change Assessment [24]). However, the OSC is the gold-standard in public sector settings in the United States and measures organizational culture and climate, two variables that are critical in our causal model. The OSC has national norms and can be used to create organizational profiles that are associated with organizational functioning. Further, the OSC has strong psychometric properties, including confirmation of the measurement model, and acceptable to high reliability on responses, moderate to high within system agreement, and significant between system differences [25].

### Implementation climate

#### Implementation Climate Assessment (ICA)

The ICA [26] is a 57-item scale that measures implementation climate that assesses the following constructs: educational support for EBTs, agency focus on EBTs, program focus on EBTs, agency recruitment of staff for EBTs, program selection of staff for EBTs, recognition for EBT use, rewards for EBT use, staff acceptance of EBTs, and supporting staff use of EBTs. Initial psychometrics are strong with good face validity and alphas in the .88 to .94 range, suggesting adequate reliability [26]. No other measures exist in mental health services to measure implementation climate, a construct first identified as an important predictor of implementation in the business literature [12].

### Leadership

#### Multifactor Leadership Questionnaire (MLQ)

This is a measure that assesses transformational leadership in organizations and asks individuals to report on the extent to which the executive and clinical directors engage in specific leadership behaviors. The MLQ will be administered separately for each of the leaders (*i.e*., executive and clinical directors); therapists will report on their leaders, and leaders also report on their own behavior. The MLQ [13] is a widely used measure that is validated across national and international settings and industries, including public sector services. The MLQ is the gold-standard tool to measure transformational leadership from the organizational literature, and psychometric analyses have confirmed the factor structure of the measurement model [27].

### Attitudes

#### Evidence-Based Practice Attitude Scale (EBPAS)

The EBPAS [28] is a well-validated, 15-item self-report questionnaire that assesses constructs related to implementation of EBTs: appeal, requirements, openness and divergence. We selected the EBPAS because it is one of the most widely used measures in implementation science, and its psychometrics are very strong. The EBPAS demonstrates good internal consistency, subscale alphas range from .59 to .90 [29], and its validity is supported by its relationship with both therapist-level attributes and organizational characteristics [30].

### Knowledge

#### Knowledge of Evidence-Based Services Questionnaire (KEBSQ)

The KEBSQ [31] is a 40-item self-report instrument to measure knowledge of the common elements of EBTs. We selected it because it is the only knowledge questionnaire that has any psychometric data suggesting its reliability and validity in assessing knowledge of common elements of EBTs, specifically temporal stability, discriminative validity, and sensitivity to training [31].

The OSC, ICA, and MLQ will be aggregated across clinicians from each agency to create organizational-level constructs if exploration of the data supports this (*i.e*., concordance between reporters).

### Dependent variables: implementation (fidelity and training). See Aim 1

#### Aim 3: to qualitatively delineate the implementation process for a subset of CMHPs

Through Aims 1 and 2, we will quantitatively estimate response to a policy on implementation of EBTs and moderators of implementation. Activities under Aim 3 will result in qualitative data from a subset of agencies to understand key informants’ perspective about the implementation process, and will generate information about the mechanisms through which organizational- and leader-level variables may drive implementation [15,32]. We will use qualitative methods to expand and more deeply understand quantitative findings from Aims 1 and 2. We will use a purposive sampling strategy [33] to identify individuals who will participate in semi-structured interviews at 2 CMHPs that are high-performing, 2 that are average-performing, and 2 that are low-performing. The interviews will explore the views and perspectives of executive directors and clinicians regarding their experience with the implementation process and their understanding of organizational and leader factors that impacted implementation. This partnership with executive directors, clinical directors, and clinicians will be valuable in interpreting quantitative findings and to provide support for or against the causal model outlined.

#### Selection of sites and key informants

Following the second measurement point in year three, the 30 sampled CMHPs will be ranked to identify the highest-, average- and lowest-performing sites in terms of fidelity gathered in Aim 1. This ranking will be continuous as based on average total score on the TPC-R per CMHP. We will visually inspect the distribution to identify the cut-points for high-, average- and low-fidelity agencies. Based on the numerical distribution of TPC-R scores, we will create three categories: high, average and low. We will randomly select two agencies from each category to engage in qualitative inquiry. In those CMHPs, the participants will be composed of one executive leader, one clinical director, and at least four clinicians who participated in Aims 1 and 2.

#### Interviews

We will conduct interviews with the executive director and clinical director at each of the selected CMHPs (n = 6) for a total of 12 individual interviews. We chose to elicit the views and perspectives of these key informants through individual interviews because of their level of authority, and because at each site they are the sole person filling this job role. We will also conduct interviews with at least 4 clinicians at each of the selected CMHPs (n = 6) for a total of 24 interviews. We selected interviews, rather than focus groups, for the front-line clinicians because of the potentially sensitive nature of the topics to be discussed, namely job performance.

#### Interview process

We will develop an interview protocol with our qualitative expert (FB). The standardized interview guide will be structured using our theoretical model and will ensure uniform inclusion and sequencing of topics across interviews to allow for valid comparison across interviews and sites. The interview guide will have three parts. The first part will cover general views about EBTs, including: what constitutes an EBT; perceptions about why EBTs are important; perceptions about leader and provider impressions of implementation; perceptions about challenges and facilitators to implementation; beliefs about organizational- and leadership-level drivers of implementation; and implementation strategies used. In the second part of the interview, we will ask about specific EBTs implemented at that CMHC. We will ask about the organizational and leader responses to implementation of these specific EBTs and factors that facilitate or hinder implementation of these specific EBTs. In the third section of the interview, we will provide respondents with findings from the quantitative data that reflect how their provider agency is performing relative to other CMHPs on implementation and ask for their reflections on these quantitative findings.

#### Qualitative analysis

Interviews will be digitally recorded with the participants’ permission, professionally transcribed, and loaded into NVivo 10.0 software for data management and analysis. Analysis will be guided by grounded theory, which provides a rigorous, systematic approach to collecting and analyzing qualitative data and has been shown to produce robust theoretical models of social behavior in healthcare settings [34]. This approach uses an inductive process of iterative coding to identify recurrent themes, categories and relationships in qualitative data. A comprehensive coding scheme is developed based on a close reading of the text. A coding dictionary is developed that includes specific definitions of each code and criteria for good examples of code applications. Codes are applied to the data in order to tag text, which is then used in computer queries that produce fine-grained descriptions of the role of organizational- and leader-level characteristics on implementation. Every three months, we will double code a subset of 25% of all transcripts and use the inter-rater reliability function in NVivo to identify discrepancies in coding. Any disagreements in coding will be resolved through discussion.

#### Mixed methods analysis

We have elected to use mixed methods to integrate findings from Aims 1, 2 and 3. The taxonomy of the design is as follows: the structure is sequential (we will gather quantitative data prior to qualitative data and weigh them equally QUAN → QUAL); utilizing the function of complementarity (to elaborate upon the quantitative findings to understand the process of implementation as experienced by stakeholders); and the process is connecting (having the qualitative data set build upon the quantitative data set) [35].To integrate the quantitative and qualitative methods, we will follow the recently released U.S. National Institutes of Health (NIH) guidelines for best practices in mixed methods [36].

We plan to use mixed methods in two ways. First, we will use findings from the quantitative data to identify patterns in the qualitative data. To do this, we will enter quantitative findings into NVivo as attributes of each participant. These quantitative attributes will be used to categorize and compare important themes among subgroups. For example, we will enter fidelity scores into NVivo at the individual clinician level and categorize clinicians into three groups: low fidelity, average fidelity, and high fidelity. Then, if leadership support emerges as a theme from the interviews, we can query instances when leadership support is discussed with low, average, and high fidelity providers, allowing the investigative team to identify patterns and make interpretations across these groups based on quantitative categorization. Second, given that we will have collected quantitative data prior to qualitative data, if there are findings that necessitate explanation (*e.g*., attitudes towards EBTs are very low at a subset of agencies), then we can use the qualitative interviews to provide answers to unexplained quantitative results (*i.e*., expansion on quantitative findings).

#### Trial status

Study procedures have been approved by the City of Philadelphia Institutional Review Board and the University of Pennsylvania Institutional Review Board. We have just begun recruitment and data collection for the first wave of data at the time of submission of this manuscript (February, 2013).

## Discussion

### Innovation

This study contains two important innovations. First, this will be one of the first studies to prospectively follow changes in implementation of child EBTs in multiple agencies over multiple years following a policy mandate; previous studies have been largely retrospective with few data-points [4] or have focused on one agency [37]. Baseline data will be collected prior to implementation of the new policy, which allows for the power of a prospective and longitudinal design. There are a number of benefits to utilizing a prospective design, most prominently that the investigative team will be able to study the process of implementation in real-time rather than retrospectively, as most studies have done. Further, this design allows for demonstrating the temporal sequence between the policy and the resulting outcomes. This study has the potential to impact future policy mandates around implementation of innovation in urban CMHPs.

Second, this study is innovative because it will identify if organizational and leadership constructs predict implementation success, and if these constructs change as a function of implementation. To date, much implementation research has relied on cross-sectional methods to explore differences in organizational variables across agencies, making it difficult to determine causality. This study will allow the research team to identify whether the implementation process itself has an impact on organizational- and leader-level constructs.

### Limitations

This study is one of the first to investigate the impact of a policy on implementation of EBTs for youth in the public sector, and is not without limitations. Experimental manipulation would have provided a more rigorous design for assessing causality. For example, assigning half the clinics to the policy mandate, and half the clinics to no change in their practice would be a stronger design. However, the realities of a real-world public system make this design infeasible. Nevertheless, the opportunity to assess this policy mandate across an entire large service system provides an important opportunity for understanding implementation process and outcomes across the outer and inner context. Additionally, therapist turnover may result in attrition in the sample over the five-year longitudinal study. Statistical consideration of how to deal with high attrition will be necessary. For example, analyses may need to utilize cross-classified random effects statistical models to address this concern. Finally, observation of therapist in-session behavior may prove more challenging than collecting self-report data, as evidenced by previous research studies. However, members of the investigative team are experienced in fidelity assessment in public sector service settings *e.g.*, [38].

### Impact

This study has the potential to impact public health by increasing our understanding of implementation of EBTs in public sector mental health settings, a setting where traditionally underserved and vulnerable youths receive needed mental health care. Findings from this study will have the potential to inform future policy mandates around EBT implementation. Findings will also add to the implementation science literature by providing information on the impact of policy on implementation of EBTs and the potential moderating effect of organizational- and leader-level variables on implementation. The study also has the potential to improve the quality of care to youth served by the public sector by increasing the number of youth who can access quality evidence-based psychosocial treatment, and reduce the research-practice gap. This type of work will be especially important going forward given that many U.S. states, counties and cities are already either incentivizing or mandating implementation of EBTs (*e.g*., California, New York State), and the Affordable Care Act of 2014 will mandate implementation of evidence-based treatments in healthcare.

## Abbreviations

CBH: Community Behavioral Health; CMHPs: Community Mental Health Providers; DBHIDS: Department of Behavioral Health and Intellectual DisAbility Services; EBPAS: Evidence-Based Practice Attitudes Scale; EBTs: Evidence-based Treatments; EPIC: Evidence-Based Practice and Innovation Center; EPIS: Exploration, Preparation, Implementation, and Sustainment Model; ICA: Implementation Climate Assessment; KEBSQ: Knowledge of Evidence-Based Services Questionnaire; MLQ: Multifactor Leadership Questionnaire; NIH: National Institutes of Health; OSC: Organizational Social Context; QUAN/QUAL: Quantitative/Qualitative; TPC-R: Therapist Procedure Checklist-Revised.

## Competing interests

GA is an Associate Editor of Implementation Science. However, all decisions on this paper will be made by another editor. The authors declare that they have no other competing interests.

## Authors’ contributions

RB is the principal investigator for the study protocol. RB generated the idea and designed the study, was the primary writer of the manuscript, and approved all changes. DM is the primary mentor for RB’S K23 award, which provides support for all study activities. Authors GA, FB, AE, TH, KH, SM and SS are consultants on the K23 award and have provided input into the design of the study. LW is the research specialist coordinating the study and has contributed to the design of the study. All authors reviewed and provided feedback for this manuscript. The final version of this manuscript was vetted and approved by all authors.
